# Anxiety level among newly hired nurse in a specialized oncology hospital: An observational study

**DOI:** 10.12688/f1000research.74420.1

**Published:** 2022-02-09

**Authors:** Ahmad Alhroub, Hebah Albakri, Hedaia Al-Awaysheh, Aladeen Alloubani

**Affiliations:** 1 King Hussein Cancer Center, Amman, Jordan

**Keywords:** Anxiety Level, Registered Nurses, General Nursing Orientation, Specialized Oncology Hospital

## Abstract

**Background: **Anxiety is common among oncology nurses due to the complexity of oncology patients' needs and demands. The current study aimed to assess the anxiety level among newly hired nurses in a specialized oncology hospital throughout their initial period of employment, deploying a General Nursing Orientation (GNO) and the Clinical Resource Nurse (CRN) role.

**Methods:** A longitudinal one-group repeated measure design was used. Participants' demographics sheet and Sate-Trait Anxiety Inventory for Adults questionnaire were used. A total of 181 newly hired nurses participated in the study.

**Results:** The anxiety level among newly hired oncology nurses was (mean=38.65, SD=9.58) at the beginning of GNO, and the level of anxiety was highest after 90 days of employment (mean=45.71, SD=7.20). The level of anxiety among newly hired oncology nurses increased gradually from day one of the GNO, the last day of GNO, and finally, after 90 days of employment.

**Conclusions:** Nurses working in oncology workplaces face anxiety. It is important to seek nursing administrators' attention to apply proper strategies to decrease the anxiety level among newly hired nurses to help them smoothly fit into a new team to ensure safe patient care.

## Abbreviations

ANOVA: Analysis of variance

CRN: Clinical Resource Nurse

GNO: General Nursing Orientation

HAS: Hamilton Anxiety Scale

HCAC: Health Care Accreditation Council

IDRAAC: Institution for Development Research Advocacy and Applied Care

JCI: Joint Commission International

SPSS: Statistical Package for the Social Sciences

## Introduction

Anxiety is defined as “something felt, an emotional state that involved feelings of apprehension, tension, nervousness, and worry followed by physiological arousal” ( Freud, 1977). Freud stated that anxiety is an adaptive behavior that helps individuals cope with stressful situations. Intense anxiety is commonly associated with many psychiatric diseases. Cattell highlighted the types of anxiety, including emotional state and personality trait anxiety ( Cattell, 1966).

A systematic review identified 87 studies published between 1980 to 2009 across 44 countries found that the global prevalence of anxiety rate after adjusted methodological differences was 7.3% ( Baxter *et al.*, 2013). Another systematic review conducted by the Institution for Development Research Advocacy and Applied Care (IDRAAC) reviewed epidemiological anxiety disorders studies among Arab countries; the results revealed that anxiety disorder is common among the participants. Therefore, they concluded the importance of having studies about anxiety disorder and its impact on individuals and communities ( Tanios *et al.*, 2009). Several studies assessed anxiety rates amongst some Arab populations; the results showed that the anxiety rate in Saudi Arabia was 16%, 16.7% in Lebanon, and 28.2% in Jordan ( Karam *et al.*, 2008; Tanios *et al.*, 2009).

Anxiety is common among healthcare professionals, including nurses ( Chen *et al.*, 2016; Cheung *et al.*, 2016; Creedy *et al.*, 2017; Huang *et al.*, 2018; Wang & Zhang, 2017). Nurses represent a large portion of healthcare professionals; nursing is a stressful profession by its nature ( Roberts & Grubb, 2014). The workload, caring for sick patients, and work-related conflict with other healthcare providers are a few factors that cause nursing anxiety ( Jafari *et al.*, 2018). Moreover, the demand related to the tasks nurses perform at different shifts, the types of patients, and the lack of resources cause nursing job-related anxiety ( Khodadadi *et al.*, 2016; Shen *et al.*, 2016).

Anxiety is also more evident among oncology nurses; they may encounter more job-related anxiety and depression ( Hegney *et al.*, 2014; Karanikola *et al.*, 2016) due to complex treatment and patient conditions ( Nia *et al.*, 2016; Rodrigues & Chaves, 2008). Anxiety among this specific population needs more emphasis, especially newly hired nurses in oncology settings ( Karanikola *et al.*, 2016). Newly hired nurses exposed to the oncology environment would have more anxiety and may lack proper stress coping skills ( Hinds *et al.*, 1994; Rodrigues & Chaves, 2008; Wazqar, 2019; Wazqar *et al.*, 2017).

The nursing induction program is vital to prepare newly hired nurses to smoothly fit into a new working environment and enable them to provide safe patient care. Applications to several accreditation agencies at the national and international level are required, such as the Health Care Accreditation Council (HCAC), Joint Commission International (JCI), and Magnet accreditation. Yet, the anxiety level of newly hired nurses in the oncology setting is not studied well. Thus, the findings of this study will add valuable information about the anxiety level among newly hired oncology nurses.

Anxiety among nurses is a common problem and has been studied extensively worldwide ( Chen *et al.*, 2016; Cheung *et al.*, 2016; Creedy *et al.*, 2017; Glazer & Gyurak, 2008; Huang *et al.*, 2018; Wang & Zhang, 2017; Xianyu & Lambert, 2006). One study assessed the prevalence of anxiety and other mental disorders among 102 clinically active Australian nurses who work at hospitals showed that 41.2 % of nurses had anxiety ( Maharaj *et al.*, 2019). In China, a study conducted in seven governmental hospitals in one city found that the anxiety prevalence rate among Chinese nurses was around 43.4% ( Gao *et al.*, 2012). Another study was conducted in the Czech Republic, comparing anxiety prevalence between general nurses and Intensive Care Unit (ICU) nurses at three different hospitals found that 44% of general nurses had anxiety. Simultaneously, the prevalence of anxiety among ICU nurses was 28% ( Janda & Jandová, 2015). Furthermore, Taghinejad et al. assessed 86 Iranian nurses working in the mental health sector at three different general hospitals; they found that 16% of nurses had an anxiety symptom ( Taghinejad *et al.*, 2014).

Hospital settings are a very stressful working environment for healthcare professionals, especially for front-line nurses. A study that examined nursing workplace stressors at different hospitals found that increased work demands, caring for dying patients, and conflict with other healthcare providers are common sources of nursing work-related anxiety ( Jafari *et al.*, 2018; Khodadadi *et al.*, 2016). In another study, Janda and Jandová (2015) performed specific tasks, types of patients, and the increasing work demands were the causes of nurses' work-related anxiety. Moreover ( Saquib *et al.*, 2019), studied the anxiety and stress prevalence among non-Saudi foreign nurses associated with job dissatisfaction; the results showed that the anxiety level was significantly associated with the workload.

A study was conducted to assess anxiety intensity symptoms of Greek oncology nurses using the Hamilton Anxiety Scale (HAS), and it showed that 11% of oncology nurses had a moderate intensity of anxiety symptoms ( Karanikola *et al.*, 2016). Furthermore, in Iran, at a teaching hospital, Molavynejad *et al.* (2019) investigated the relationship between burnout level and personality traits among 106 oncology nurses; the results revealed that 32.1% of the oncology nurses had severe burnout with a significant positive correlation with anxiety ( Molavynejad *et al.*, 2019).

Newly hired oncology nurses may lack proper anxiety and stress coping skills ( Hegney *et al.*, 2014). Therefore, it is crucial to assess the anxiety level among newly hired oncology nurses and implement intervention programs to prepare them for the new working environment safely. Many studies have been conducted to explore GNO's effect on improving newly hired nurses' skills and knowledge ( Cockerham *et al.*, 2011; Woolwine *et al.*, 2019). In one study, Cockerham *et al*. (2011) evaluated the average of the pretest scores was 66% and after completing the program, the average score increased to 92%. GNOs also showed that newly hired nurses were successfully integrated into the hospital team ( Bahouth & Esposito-Herr, 2009).

Furthermore, other researchers conducted a literature review focused on the effect of the GNO program versus the one-year residency program on nurses' retention and turnover. They found that newly-graduated nurses' satisfaction and retention were higher with the one-year residency program versus the orientation program alone ( Eckerson, 2018). Nurses reported improvements in their perception of patient care quality when a senior CRN accompanied them. A quality improvement project, conducted at a labor and delivery floor in an academic medical center to assess the efficacy of the CRN role in supporting units with high percentages of new-to-practice staff, found that the perception of quality patient care increased from 16 % pre-implementation of the CRN role to 50% post-implementation ( Maloney & Nelson, 2013).

Another study was conducted to assess anxiety changes among newly hired nurses at different points in time during their first year of employment; the results indicated that the anxiety level was 12.21 after one week of the residency program to 14.17 after one month and 15.30 at the end of the second month ( Lin *et al.*, 2020).

The current study aimed to assess the anxiety level among newly hired nurses in a specialized oncology hospital throughout their initial period of employment, deploying a GNO and the CRN role.

## Methods

### Design

A longitudinal one-group repeated measure design was used in the study ( Salkind, 2012). In a longitudinal study in which progress over time is measured, repetitive observations are gathered ( Polit & Beck, 2016).

### Sampling

The study used purposive sampling of newly hired registered nurses, with inclusion criteria of having at least a bachelor's degree, attending the GNO, and provision of nursing care directly to patients with cancer. The purposive sample was used as we sought a selection with particular criteria that helped the study purpose ( Patton, 2005).

The anticipated sample was calculated using G*-POWER with a consideration of having an alpha of .05, a power of .80, a medium effect size (d = 0.25) (Cohen, 1988), which resulted in a sample of 128 participants. Oversampling was intended to overcome the attrition rate if the participants could not be reached or if there was an incomplete questionnaire. So, a total of 200 participants were selected.

### Setting

This study was conducted at a specialized oncology hospital, a tertiary cancer center in Jordan. It receives more than 3500 newly diagnosed oncology patients every year. This hospital is equipped with advanced technology, medical equipment, and services, comprising 352 beds, including two ICUs, one for adults and one specialized for pediatrics. Oncology care is provided by qualified oncologists and healthcare experts, such as trained nurses, especially in oncology care nursing, who work collaboratively to guarantee that patients receive safe and advanced cancer care.

### Instruments

The State-Trait Anxiety Inventory for Adults™ (STAI-AD) is a reliable tool for assessing adults' anxiety levels. The tool is translated into many languages. The internal consistency mean was .89 for the trait scale and .91 for the state scale. The test-retest reliability mean for the trait scale was .88. As expected, the state scale mean was lower than .70, indicating the transitory nature of the measure ( Barnes *et al.*, 2002). Moreover, Spielberger *et al.* (1999) have displayed good concurrent validity, as scores on the STAI correlate extremely with findings observed on alternative anxiety measurement tools such as the Anxiety Scale Questionnaire.

Five experts assessed the content validity of the STAI-AD, determining if the items correctly reflected the STAI characteristics. A psychologist, an oncology nurse, a psychiatrist, a nurse manager, and a psychosocial specialist were among the experts. In order to determine the extent of relevancy to the newly hired nurses, experts were asked to evaluate each item in the tool. The scoring system was a scale from 1 to 4 (4 = very relevant, 3 = relevant, 2 = slightly relevant, 1 = not relevant to anxiety level among newly hired nurses).

The two STAI subscales are performed and scored from one to four. Participants apply a 4-point Likert scale for the state part to reflect how certain items about anxiety refer to the individual at a specific right moment. Likert scales range from 1 (“not at all”) to 4 (“very much so”). The trait part provides a comparable 4-point scale but directs how participants feel in general. The Likert scale for this part ranges from 1 (“almost never”) to 4 (“almost always”). Scores are calculated to give a total score for each subscale.

Every item in the STAI is given a weighted score of 1 to 4. A score of 4 shows the presence of a high anxiety level such as “I feel upset” and “I feel frightened.” Also, the scoring weights for the anxiety-negative items were reversed. We added the weighted scores for the 20 items on each scale. Therefore, scores for both the State-Anxiety and the Trait-Anxiety scales can vary from a minimum of 20 to a maximum of 80.

“This instrument is covered by the U.S. and international copyright laws as well as various state and federal laws regarding data protection. In whole or in part, any use of this instrument is subject to such laws and is expressly prohibited by the copyright holder. If you would like to request permission to use or reproduce the instrument, in whole or in part, contact Mind Garden, Inc.” (Spielberger *et al.*, 1999).

The concepts of state and trait anxiety were often used in literature; basically, personality states refer to what individual feeling and emotional reactions at this moment of time induced by stressful experimental procedures such as starting a new job. Subjective individualized feelings would reflect anxiety while personality traits indicate individuals *generally* feel.

### Data collection procedure

Data collection included participants' demographics sheet and the STAI-AD. Data were collected on day one of the GNO, upon completion of GNO, and after 90 days of the intensive unit-based orientation program with clinical resource nurses (CRN). All newly hired nurses were trained to provide safe, optimal, and excellent patient care through a nursing induction program consisting of a GNO program followed by a mentorship period with a CRN. A CRN is responsible for administering clinical expertise to newly hired nurses. The CRN is qualified to monitor and help the nursing team to provide nursing care based on best practices. Clinical activities may include direct care to the patients and coordination with the healthcare team.

The nursing induction program is specially developed to prepare newly hired registered nurses to work safely and independently. It includes identifying the organization's values, mission, vision, and objectives, which would help the nurses enroll in the new environment smoothly. However, it also stresses vital patient safety issues, a standard of patient care and provides core mandatory training and competencies required to begin their new job.

The GNO program extends over ten working days and scheduled quarterly data collected from May 2018 to Aug 2019. All newly hired nurses attend a series of lectures focused on patient safety, primary nursing care, stress management, and advanced oncology care. Additionally, a three-month mentorship program with a CRN at the unit level would be completed to ensure that nurses accomplish all required competencies. Our hospital induction program was created using a competency-based assessment method, “Novice to expert clinical practice model” ( Benner, 1984). The activity-based teaching/learning strategies used in the GNO program support learners' combined knowledge with proper clinical situations.

### Ethical consideration

Ethical approval to conduct this study was received from the Institutional Review Board (IRB) at King Hussein Cancer Center. All methods were performed in accordance with the relevant guidelines and regulations. The researchers obtained an institutional review board approval to conduct the study; the approval code is 18KHCC36.

The participants were met in a private room to explain the study's goals and discuss consent to participate in the study. We explained and described the study's purposes and the research outcomes for each participant. Then, the researcher(s) asked the participants to sign the consent form. All forms were numerically coded before being given to the participants. RNs were provided envelopes to put the completed forms to ensure anonymity and confidentiality. No particular participant information could be associated with any response.

The questionnaire and the STAI-AD were administered through a paper and pencil survey. Newly hired registered nurses voluntarily filled out the survey at the beginning of the GNO and upon completion, as well as after 90 days of the GNO.

Study design, sampling, and selection were determined in the initial planning stage of a study to reduce any potential source of bias. Moreover, 19 participants were excluded from the study and analysis due to incomplete outcomes assessment.

### Data analysis

Data were analyzed using Statistical Package for the Social Sciences (SPSS) version 21. Descriptive and inferential statistics were used to meet the study's aims at a significance level of .05. Descriptive statistics were used to describe demographic characteristics based on the level of measurements. One-way repeated measured analysis of variance (ANOVA) was used to compare the anxiety level at different periods of time.

## Results

### Demographic characteristics

Questionnaires were distributed to 200 nurses; at the beginning of the GNO, 200 participants returned the questionnaire with a response rate of (100%). Upon completion of the GNO, 188 participants returned the questionnaire with a response rate of (94%), and after three months of the CRN role, 181 returned the completed questionnaire (90.5% response rate). Demographic data showed that 64.6% of participants were female, 93.9% single nurses; most of the participants had a bachelor in nursing degree and had Jordanian nationality (n=180, 99.4%). The mean of participants' age was 23.30 years (SD=3.52), while the average of their past experience years was 1.02 (SD=1.01) ( Table 1).

**Table 1.** Demographic data.

Variable		Frequency	%
Gender	Male	64	35.4
	Female	117	64.6
Marital Status	Single	170	93.9
	Married	11	6.1
Educational	Bachelor	180	99.4
	Master	1	0.6
Nationality	Jordanian	180	99.4
	Non-Jordanian	1	0.66

	Mean	Std. deviation
Age	23.30	3.52
Experience	1.02	1.01

### Anxiety level

The study results revealed that the anxiety level among the nurses was lowest in their general daily life. However, at the beginning of the study, the mean anxiety level was 39.2 (SD=8.61); and, the mean of anxiety among participants was highest after 90 days of employment ((45.71, SD=7.20). The participants' anxiety level increased gradually from the initial contact at day one of the GNO, then on to the last day of GNO, and finally after 90 days of employment ( Table 2).

**Table 2.** Anxiety level.

	N	Minimum	Maximum	Mean	Std. deviation
General anxiety	181	20.00	75.00	38.65	9.58
Period 1	181	20.00	63.00	39.20	8.61
Period 2	181	23.00	62.00	44.20	7.82
Period 3	181	23.00	57.00	45.71	7.20

ANOVA was used to evaluate the change in participants' level of anxiety when measured before participating in the GNO, on the last day of GNO, and after 90 days. The results of the repeated measure ANOVA revealed a significant time effect, Wilks’ Lambda=.668, *F* (2, 179)=44.47, *p*>.001, *n* ^2^=.332. Follow-up comparisons revealed that each pairwise difference was significant, *p*>.001 ( Table 3). So, there was a significant increase in anxiety levels over time.

**Table 3.** F tests the multivariate effect of anxiety.

Anxiety	Mean	Std. error	95% Confidence interval	
			Lower bound	Upper bound
Period 1	39.203	.640	37.940	40.467
Period 2	44.205	.581	43.057	45.352
Period 3	45.717	.536	44.660	46.774

	Value	F	Hypothesis df	Error df	Sig.	Partial eta squared	Noncent. parameter	Observed power ^b^
Wilks' lambda	.668	44.474 ^a^	2.000	179.000	.000	.332	88.949	1.000

^a^ Exact statistic.

^b^ Computed using alpha=.05.

The Cronbach Alpha Reliability was .893 and .865 for STAI Y1 (Present feeling) and Y2 (General feeling), respectively ( Table 4).

**Table 4.** Cronbach alpha reliability statistics.

Questionnaire type	Cronbach's alpha	N of items
STAI Form Y1 (Present feeling)	.893	20
STAI Form Y2 (General feeling)	.865	20

## Discussion

The current study's primary objective was to assess the anxiety level among newly hired oncology nurses at three different times during the nursing induction program. A large portion of nurses included in this study were newly graduated nurses.

This study's results indicate an increase in the anxiety level among newly hired nurses in the oncology hospital from the beginning of employment to the end of the induction program. Overall, the findings revealed that newly hired oncology nurses in this study had different levels of anxiety. This finding is consistent with previous studies, which showed that nurses reported anxiety ( Karanikola *et al.*, 2016; Molavynejad *et al.*, 2019). Compared to other nursing specialties, a study was conducted among Critical Care nurses in Greece, which showed that around a quarter of participants reported moderate to severe anxiety symptoms ( Karanikola *et al.*, 2012). In another study conducted in China, almost 43.4% of nurses experienced anxiety ( Gao *et al.*, 2012), which was also consistent with another study conducted among nurses in Iran ( Kayalha *et al.*, 2013). Other nursing studies reported a high to moderate anxiety levels among non-oncology nurses worldwide ( Chen *et al.*, 2016; Cheung *et al*., 2016; Creedy *et al.*, 2017; Glazer & Gyurak, 2008; Huang *et al.*, 2018; Wang & Zhang, 2017; Xianyu & Lambert, 2006).

The study findings indicate that newly hired oncology nurses' anxiety levels were consistently increasing from the beginning of the GNO program to the end of the GNO, and three months period subsequently. These results are similar to the findings of Lin *et al.* (2020); who reported that anxiety level reaches the peak after three months of employment among newly hired nurses despite enrolling them in a well-structured nursing residency program ( Lin *et al.*, 2020). This result could be viewed in the context of our nursing induction program schedule and timetable as the first ten days of the GNO program include class-based lecturing away from the clinical setting. These lectures focus primarily on general concepts related to policy, procedure, and oncology patient care; therefore, the newly hired nurses' anxiety level is low. However, as the induction program progressed, the workload of newly hired oncology nurses increased gradually to meet the orientation program expectations, which led to increasing the nurse's anxiety.

Other studies also revealed that having a complex relationship with other healthcare providers was a factor causing work-related anxiety among nurses in the oncology setting ( Escot *et al.*, 2001; Isikhan *et al.*, 2004). The direct supervision role performed by the senior CRN could contribute to increasing newly hired nurses' anxiety in the clinical setting, along with the fact that they have to pass all required competencies to become independent practitioners. Additionally, by the end of three months of employment, a probationary evaluation would increase the levels of anxiety of the newly hired nurses. Most participants were new graduate nurses, young with little previous clinical experience, which meant a lack of proper stress coping skills. Similarly, Hegney *et al.* in 2014 showed that anxiety levels are higher among younger nurses, a full-timer, and without specific postgraduate qualification and training ( Hegney *et al.*, 2014).

Another explanation of the current study findings that, in oncology hospitals, nurses are looking after patients who require frequent nursing care; the workload increases gradually as the program goes on, which leads to an increase in nurses' anxiety. Additionally, nurses working in oncology settings might encounter more job-related anxiety and depression due to the type of medical situations such as treatment complexity and patient conditions. Keskin *et al.* found that workload and an increasing number of patients lead to anxiety and depression among oncology nurses ( Keskin *et al.*, 2018). Subsequently, nurses witness the patients' suffering, family distress, and possible death at a certain disease progression stage. In such situations, newly hired oncology nurses' ability to cope with end-of-life situations influences the oncology nurse's death anxiety level.

The unavailability of a psychological support intervention for newly hired oncology nurses during the hospital induction program could explain the current study findings. The program focused on improving nurses' knowledge and skills through the oncology training course and introducing the end-of-life concept. The absence of psychological support intervention flags the importance of how to support newly hired oncology nurses once they are exposed to stressful and unpleasant situations. Several studies highlighted the importance of integrating psychological support intervention in clinical environments such as art and music therapy ( Doo *et al.*, 2018), using emotional intelligence as anxiety tool management ( Kadda, 2014), mindfulness-based programs ( Foureur *et al.*, 2013), and guided imagery ( Boehm & Tse, 2013).

The newly hired nurses in this study had anxiety despite completion of the nursing induction program; limited literature looked at the effect of GNO and mentorship program by CRN on newly hired nurses at the oncology center. Our results are consistent with Cockerham *et al.*, who investigated the effect of GNO and mentorship programs on improving newly hired nurses' knowledge and skills; they found that the average of the pretest scores was 66%, and after completing the program, the average score increased to 92%. Hence Lin *et al.* (2020) focused on exploring changes in anxiety level and work stress among newly hired nurses over the two-year residency program. They conclude that anxiety reached its peak at the first three months of employment and then stabilized until the end of the program. The current study results provide baseline data for the further nursing study. Furthermore, the organization invests in human resource development creating clinic-oriented educator role such as CRN to facilitate unit-based orientation programs and mentorship. Thus, this would ease the new nurses' enrollment and decrease the anxiety associated with the new role and/or environment.

The current study has clinical implications and suggestions for planning interventions to assist oncology nurses in reducing levels of anxiety. Nurse leaders, managers, and educators should create new interventions such as support groups, physical and mental exercises, counseling support, and anxiety control sessions, motivating nurses to verbalize their feelings to help nurses completely control their own anxiety ( Aycock, 2009; Henry, 2014). Oncology hospitals should provide healthy working environments and create particular interventions to decrease nurses' stressors. Given these results, anxiety-related work should be addressed and controlled to avoid growing tension among nurses and support nurses during the first phase of employment to reduce or eliminate anxiety.

### Limitation

The study was conducted at a specialized oncology hospital in Jordan. The cross-sectional design can limit the inferences of causality ( Brady Germain & Cummings, 2010). Moreover, the study was limited to assessing the oncology nurses' anxiety level; the investigators didn't determine the intensity of anxiety for nurses. Given the standard procedure to conduct a GNO to newly hired nurses, assessing its impact versus not receiving a GNO (control group) without compromising patient care on anxiety levels is difficult. Further studies are recommended to evaluate the severity of anxiety for this target population more accurately.

## Conclusion

Nurses working in oncology workplaces frequently encounter several anxiety conditions in their settings, leading to psychosocial and physical problems. So, nurses should be willing to care for themselves to maximize the optimization of their health and to decrease anxiety at work; on the other hand, seeks Nursing administrator attention to address this phenomenon and modify nursing induction programs in order to apply proper strategies to reduce anxiety level among newly hired nurses also help them to fit into a new team smoothly to ensure safe patients care.

## Data availability

### Underlying data

Harvard Dataverse: “Anxiety Level among Newly Hired Nurse in a Specialized Oncology Hospital”, https://doi.org/10.7910/DVN/OSXHWE ( Alloubani, 2021).

This project contains the following underlying data:

- Anxity research - Statistics.tab

Data are available under the terms of the Creative Commons Zero “No rights reserved” data waiver (CC0 1.0 Public domain dedication).
