# Supplementary material for: Anxiety level among newly hired nurse in a specialized oncology hospital: An observational study
Source: F1000Res. 2022 Feb 9;11:162. [Version 1] doi: 10.12688/f1000research.74420.1 (PMC8961193; doi:10.12688/f1000research.74420.1)
Supplement: Peer review history

# Peer review: Reviewer response for version 1

**DOI:** 10.5256/f1000research.78173.r127075

**Published:** 2022-03-28

**Authors:** Elham H. Othman

**Affiliations:** 1 School of Nursing, Applied Science Private University, Amman, Jordan

Thank you for the opportunity to review this article, which addressed a significant issue among nurses working with oncology patients. The research team proficiently presented their study and findings. However, I suggest a few modifications to improve the quality and readership of the paper.

**Introduction:**

 The introduction section was well-written and comprehensive. However, I noticed redundancy in presenting previous literature, which can be re-phrased to improve the reading flow.

 In light of the study aim, I suggest modifying the following paragraphs, as these paragraphs addressed the impact of the GNO or CRN on staff anxiety, which might confuse the readers about the study aim.

- Newly hired oncology nurses… hospital team (Bahouth & Esposito-Herr, 2009).
- Furthermore, other researchers … 50% post-implementation (Maloney & Nelson, 2013).

    

**Methodology**:

 In the sampling section, you may specify the duration of data collection. As you mentioned, you collected data from 181 newly hired nurses, were they engaged in the same period?

 In the instrument section: In the second paragraph: *“Five experts assessed the content validity of the STAI-AD, determining if the items correctly reflected the STAI characteristics.” *Is that necessary, as the instrument is already validated? And suppose you mean that it was validated to determine the extent of relevancy to the newly hired nurses. In that case, I suggest modifying the first sentence and adding the results of the experts’ validation.

**Results:**

 You presented the Cronbach Alpha Reliability twice, in a paragraph and in Table 4, which is redundant. I suggest keeping the text and deleting the Table.

**Discussion:**

 You mentioned that *a large portion of nurses included in this study were newly graduated nurses*; it would be better if you added the number and percentage of these nurses in the result section.

 In the sixth paragraph, you stated: ‘ *The newly hired nurses in this study had anxiety despite completion of the nursing induction program; limited literature looked at the effect of GNO and mentorship program by CRN on newly hired nurses at the oncology center’. *This sentence implies that the study examined the effect of such programs on anxiety rather than only observing and describing the anxiety levels. I suggest modifying it according to your study aim.

 In the same paragraph, you said: ‘ *Our results are consistent with Cockerham et al., who investigated the effect of GNO and mentorship programs on improving newly hired nurses' knowledge and skills’. *However, I am not sure how that is consistent with your study. The cited study measured knowledge and skills, while you measured anxiety. I advise deleting or replacing it.

 In the Limitation section, you said ‘ *The cross-sectional design can limit the inferences of causality*,' which is irrelevant as you conducted a longitudinal study. Please correct.

Is the work clearly and accurately presented and does it cite the current literature?

Yes

If applicable, is the statistical analysis and its interpretation appropriate?

Yes

Are all the source data underlying the results available to ensure full reproducibility?

Yes

Is the study design appropriate and is the work technically sound?

Yes

Are the conclusions drawn adequately supported by the results?

Yes

Are sufficient details of methods and analysis provided to allow replication by others?

Yes

Reviewer Expertise:

Nursing research. Oncology and End of Life care.

I confirm that I have read this submission and believe that I have an appropriate level of expertise to confirm that it is of an acceptable scientific standard.

# Peer review: Reviewer response for version 1

**DOI:** 10.5256/f1000research.78173.r123036

**Published:** 2022-03-01

**Authors:** Nazih Abu Tabar

**Affiliations:** 1 Assistant Professor, Nursing Department, Fatima College of Health Sciences, Al Ain, United Arab Emirates

Thank you for the opportunity to review this article.

 In general, the study construction is very good. It is easy to read and follow. The study topic is discussing important factors that affect the nursing profession. The authors succeed in describing the problem and highlighted the importance of reviewing these issues in the nursing profession and among Jordanian nurses as the targeted country of the study population.

 The methods used in the study are a longitudinal, one-group repeated measure design. A valid and reliable questionnaire was used. Given the stated aim of the study to “assess the anxiety level among newly hired nurses through three periods of employment at the specialized oncology center. However, the main worries related to the methods are:

- The sampling method is a purposive sample, was the sample size affected by rates of refusals or attrition? if yes so it is a convenient sample method. ‎The method of calculating total sample size is not defined by which statistical test was used to decide if it is sufficient to meet the assumption of used statistical analysis test. So, was the sample size enough to support the conclusion validity? Another worry in the methodology is not clear which scale was used in the state-trait anxiety inventory questionnaire.

The Results of the study are clear and well presented. The statistical analysis seems to be appropriate given the study aim, variables, and nature of the data collected.

 The Discussion seems very good, it compared the results of the current study with similar national and international literature. It suggests the implications of the results and interventions of the study to support the newly hired nurses. The critical point in the discussion, the authors try to prove the impact of the nursing induction program which highlights some concerns about the aim of the study and methodology were used. The limitations need some revision and editing because it mismatches with study aims and methodology.

 Overall, this article includes significant information in a particular setting that can be useful in guiding efforts to promote and support an appropriate intervention to support a newly hired nurse.

Is the work clearly and accurately presented and does it cite the current literature?

Yes

If applicable, is the statistical analysis and its interpretation appropriate?

Yes

Are all the source data underlying the results available to ensure full reproducibility?

Yes

Is the study design appropriate and is the work technically sound?

Yes

Are the conclusions drawn adequately supported by the results?

Yes

Are sufficient details of methods and analysis provided to allow replication by others?

Yes

Reviewer Expertise:

Nursing

I confirm that I have read this submission and believe that I have an appropriate level of expertise to confirm that it is of an acceptable scientific standard.
